# Antimicrobial Resistant Bacteria Monitoring in Raw Seafood Retailed: a Pilot Study Focused on *Vibrio* and *Aeromonas*

**DOI:** 10.14252/foodsafetyfscj.D-23-00006

**Published:** 2023-10-14

**Authors:** Akira Fukuda, Ryu Tsunashima, Masaru Usui

**Affiliations:** Food Microbiology and Food Safety Unit, Division of Preventive Veterinary Medicine, School of Veterinary Medicine, Rakuno Gakuen University, 582 Bunkyodai Midorimachi, Ebetsu, Hokkaido 069-8501, Japan

**Keywords:** *Vibrio*, *Aeromonas*, seafood, antimicrobial resistant bacteria, antimicrobial resistance genes

## Abstract

In aquaculture, bacterial infections in sea animals are treated using antimicrobials. As seafood is frequently consumed in its raw form, seafood contaminated with water-borne antimicrobial-resistant bacteria presents a potential transmission route to humans and can influence food safety. In this study, we aimed to determine the abundance of water-borne bacteria in retail raw seafood and to characterize their antimicrobial resistance profiles. In total, 85 retail raw seafood samples (32 fish, 26 shellfish, 25 mollusks, and two crustaceans) were purchased from supermarkets in Japan, and water-borne bacteria were isolated. The isolated bacterial species predominantly included *Vibrio* spp. (54.1%) and *Aeromonas* spp. (34.1%). *Vibrio* or *Aeromonas* spp. were isolated from more than 70% of the seafood samples. Tetracycline-, sulfamethoxazole-, and/or trimethoprim/sulfamethoxazole-resistant *Vibrio* or *Aeromonas* spp. isolates were detected in seven (21.9%) fish samples (two wild-caught and five farm-raised) harboring *tet*, *sul*, and/or *dfr* genes. Sulfamethoxazole- and trimethoprim/sulfamethoxazole-resistant isolates were only detected in farm-raised fish. Tetracycline and sulfamethoxazole are commonly used in aquaculture. These results suggest that water-borne bacteria like *Vibrio* and *Aeromonas* spp. should be the primary focus of antimicrobial-resistant bacteria monitoring to effectively elucidate their spread of bacteria via seafood.

## Introduction

The emergence and spread of antimicrobial-resistant bacteria (ARB) and antimicrobial resistance genes (ARGs) have become a major public health concern. As antimicrobials use exerts a selective pressure; therefore, antimicrobials using environments are considered to be ARB and ARGs hotspots^1^^)^. In human clinical settings, livestock, and livestock-associated food production, ARB are continuously monitored among contaminating bacteria; particularly in clinical settings, coliforms, such as *Escherichia coli*, are monitored in various countries including Japan^2^^,^^3^^)^.

As antimicrobials are used to treat bacterial infectious diseases in farm-raised seafood, aquaculture is considered a hotspot for the emergence and spread of ARB and ARGs^4^^)^. Moreover, seafood consumption has increased globally, and it is frequently consumed in a raw form, particularly in Japan, where sashimi and sushi are popular dishes^5^^)^. Consequently, seafood consumption may represent an important ARB transmission route to humans.

Pathogenic bacteria, such as those that cause food poisoning, are prevalent in seafood, and bacterial contamination in fish is frequent along the food value chain^6^^,^^7^^,^^8^^)^. Notably, ARB and ARGs have been studied in seafood and related samples^9^^)^. However, most previous studies have only focused on pathogenic bacteria. In an aquatic environment, *Vibrio* and *Aeromonas* would be candidates indicator bacteria for antimicrobial resistance; *Vibrio* would be especially suitable as indicator bacteria for marine settings because of their natural habitat^10^^,^^11^^)^. Further, *Vibrio* and *Aeromonas* were usually isolated from retailed seafood^12^^,^^13^^)^. However, the species of contaminating bacteria, which usually inhabit aquatic environments, especially *Vibrio*, in retail raw seafood in Japan and the prevalence of antimicrobial resistance in these bacteria remain unknown^14^^,^^15^^)^.

This study aimed to clarify the prevalence of water-borne bacterial species, specifically *Vibrio*, in retail seafood intended for raw consumption, as well as their antimicrobial resistance profiles in Japan. This investigation also acts as a pilot study for monitoring ARB in seafood.

## Material and Methods

### Sampling of Raw Seafood

In total, 85 fresh domestic seafood samples intended for non-heated raw consumption were purchased from eight supermarkets surrounding Rakuno Gakuen University (Ebetsu, Hokkaido Prefecture, Japan), between July 2020 and September 2021. The samples were refrigerated and not frozen. The type and origin of seafoods distributed varied depending on the season, especially in wild-caught seafoods. To collect a wide variety of seafoods, 3–10 randomly selected samples were purchased every month, for more than one year. The samples comprised 32 fish, 26 shellfish, 25 mollusks, and two crustaceans originating from 10 prefectures. (Tables S1 and S2). Among fish samples, 14 were wild-caught and 18 were farm-raised. All shellfish, mollusk, and crustacean samples were wild-caught. All collected samples were refrigerated at 4°C for up to 24 h before use.

### Isolation of Bacteria

For bacterial isolation, the edible parts excluding the digestive tract were used. Twenty-five grams of each sample was shredded using sterilized scissors and placed in a sterile plastic bag containing 225 mL of alkaline peptone water supplemented with 2% sodium chloride (Nissui Pharmaceutical, Co., Ltd. Tokyo, Japan). Each sample was homogenized at 200 rpm for 1 min using a stomacher (EXNIZER-400, Organo, Tokyo, Japan). One hundred microliters of the homogenized sample was plated onto TCBS (Eiken Co. Ltd., Tokyo, Japan) and CHROMagar™ Vibrio agar (CHROMagar, Paris, France). The homogenized samples were incubated at 30°C for 24 h as a preculture; the precultures were then plated onto TCBS and CHROMagar™ Vibrio agar. The agars were incubated at 30°C for 24 h, and a maximum of 10 colonies were isolated from each sample; two colonies/color from each plate (yellow and green colonies grown on TCBS agar, as well as purple, blue, and cream-colored colonies grown on CHROMagar™ Vibrio agar) were used for further analysis.

### Bacterial Identification

The bacterial species of each isolate was determined using matrix-assisted laser desorption/ionization time-of-flight mass spectrometry on a Bruker MALDI Biotyper system (Bruker Daltonics, Bremen, Germany)^16^^)^. Additionally, PCR was performed to confirm the bacterial species in genus of *Vibrio* and *Aeromonas* (**Table S3**)^17^^,^^18^^,^^19^^,^^20^^)^. In subsequent experiments, isolates from each agar plate showing the same color and antimicrobial susceptibility profile were considered as a single isolate.

### Susceptibility to Antimicrobials

Susceptibility to antimicrobials was determined using the agar dilution method, and resistance breakpoints were defined in accordance with the Clinical Laboratory Standards Institute guidelines^21^^)^. *Escherichia coli* ATCC25922 was used as a quality control strain in CLSI M45. The following antimicrobials were tested against *Vibrio* and *Aeromonas* isolates: cefotaxime, chloramphenicol, ciprofloxacin, gentamicin, tetracycline, and trimethoprim/sulfamethoxazole. Additionally, ampicillin, cefazolin, and sulfamethoxazole were tested against *Vibrio* strains (all obtained from Sigma-Aldrich, St. Louis, MO).

### Detection of ARGs

ARGs were screened according to antimicrobial susceptibility phenotypes. Primers used for detecting ARGs are shown in **Table S4**. For tetracycline-resistant isolates, *tet* genes were detected^17^^,^^22^^)^. For trimethoprim/sulfamethoxazole- or sulfamethoxazole-resistant isolates, *sul* and *dfr* genes were detected^23^^,^^24^^,^^25^^)^.

### Statistical Analysis

The Chi-squared test was used to compare the isolation rates of bacteria among the samples. Statistical significance was set at *p*<0.05.

## Results and Discussion

### Bacterial Identification

*Vibrio* spp. (54.1%; n=46) was isolated from 85 raw seafood samples, followed by *Aeromonas* spp. (34.1%; n=29), *Shewanella* spp. (17.6%; n=15), and *Photobacterium* spp. (10.6%; n=9) (**Table 1**). The combined isolation rate for*Vibrio* or *Aeromonas* spp. was 74.1%. *Vibrio* and *Aeromonas* spp. were commonly isolated from commercially available fresh seafood intended for raw consumption in Japan. These results are consistent with previously reported bacterial isolation rates from raw seafood even though the types of seafoods and distribution channels vary from region to region^13^^)^. *Vibrio* spp. and *Aeromonas* spp. have been recognized as typically water-borne and several of their species have been identified as potential food-borne pathogens and causative agents of fish diseases^15^^,^^26^^)^. Notably, the rates of *Vibrio* spp. isolated from wild-caught shellfish were higher than those isolated from wild-caught fish. *Aeromonas* spp. was isolated at higher rates in wild-caught and farm-raised fish than in shellfish and mollusks. These variations in isolation rates are likely caused by the diversity of microbiomes associated with different types of seafood and their growing environments^27^^)^. Despite limitations of sample size and location, we demonstrated that *Vibrio* or *Aeromonas* spp. could be frequently isolated from raw seafood, sold in retail stores in Japan, consistent with their status as water-borne bacteria. However, further verification, using a larger number of samples from different regions, is necessary to confirm whether the research results are widely applicable.

**Table 1.** Isolation rates (%) of several bacterial species from retail raw seafood

Bacterial species (%)	Total	Fish		Shellfish	Mollusks	Crustacean
		Wild-caught	Farm-raised	Wild-caught	Wild-caught	Wild-caught
	(n = 85)	(n = 14)	(n = 18)	(n = 26)	(n = 25)	(n = 2)
*Vibrio* spp.	54.1	28.6	50.0	73.1	48.0	100
*Aeromonas* spp.	34.1	71.4	61.1	3.8	24.0	50.0
*Shewanella* spp.	17.6	14.3	0	10.8	24.0	0
*Photobacterium* spp.	10.6	7.1	27.8	3.1	4.0	0
*Staphylococcus* spp.	7.1	0	0	1.5	20.0	0
*Morganella* spp.	5.9	7.1	5.6	1.5	4.0	50.0
*Pseudomonas* spp.	3.5	21.4	0	0	0	0
Other	11.8	21.4	5.6	1.5	16.0	50.0
Either *Vibrio* or *Aeromonas* spp.	74.1	78.6	72.2	73.1	72.0	100

**Table 2.** Antimicrobial resistance rates of *Vibrio* spp. isolated from seafoods

Antimicrobial resistance rates (%)	Wild-caught fish (n = 13)	Farm-raised fish (n = 22)	Shellfish (n = 52)	Mollusks (n = 24)	Crustacean (n = 4)
Tetracycline	7.7	13	0	0	0
Sulfamethoxazole	0	8.7	0	0	0
Trimethoprim/sulfamethoxazole	0	4.3	0	0	0
Ampicillin	61.5	82.6	82.7	75	100

### Susceptibility to Antimicrobials

In total, 115 *Vibrio* spp. and 65 *Aeromonas* spp. were isolated and used for susceptibility to antimicrobials (Tables 2, 3, and S5). Tetracycline resistance rates in *Vibrio* spp. isolates derived from wild-caught and farm-raised fish were 7.7% and 13.0%, respectively. Tetracycline resistance rates in *Aeromonas* spp. isolates derived from wild-caught and farm-raised fish were 3.2% and 4.0%, respectively. Among *Vibrio* spp., derived from farm-raised fish, resistance rates to sulfamethoxazole and trimethoprim/sulfamethoxazole were 8.7% and 4.3%, respectively. The trimethoprim/sulfamethoxazole resistance rate in *Aeromonas* spp. was 12.5%. In many countries, including Japan, tetracycline, trimethoprim, and sulfamethoxazole derivatives are commonly used antimicrobials in aquaculture, and may serve as selective pressures for ARB^3^^,^^4^^,^^9^^,^^28^^)^. Notably, in our study, isolates resistant to sulfamethoxazole- and/or trimethoprim/sulfamethoxazole were detected only in samples derived from farm-raised fish, suggesting a possible link to the situation of antimicrobials usage in aquaculture. Moreover, the prevalence of antimicrobial-resistant *Vibrio* and *Aeromonas* spp. in seafood indicates the use of antimicrobials in aquaculture. Among all sample types, more than 60% of *Vibrio* spp. isolates displayed ampicillin resistance. Further, approximately 10% of *Aeromonas* spp. isolates from wild-caught fish, farm-raised fish, and mollusk samples displayed cefotaxime resistance. *Vibrio* and *Aeromonas* showed intrinsic resistance to these antimicrobials through chromosome-mediated resistance mechanisms^15^^,^^26^^)^. None of the *Vibrio* and *Aeromonas* spp. isolates were resistant to the other antimicrobials tested in our study. The antimicrobial resistance rates observed in our study are consistent with those reported in other countries, such as China, Norway, Thailand, and USA^13^^,^^29^^,^^30^^,^^31^^)^. Further, ARB contamination in seafood preparation could occur through various sources including human handlers^8^^,^^32^^,^^33^^)^. To qualify the ARB-mediated risk to human health via seafood, identifying suitable ARB markers capable of evaluating contamination in seafood is important^34^^)^. Therefore, additional research on the prevalence of antimicrobial-resistant water-borne bacteria, especially on *Vibrio* and *Aeromonas* spp., is required to enhance seafood safety.

**Table 3.** Antimicrobial resistance rates of *Aeromonas* spp. isolated from seafoods

Antimicrobial resistance rates (%)	Wild-caught fish (n = 31)	Farm-raised fish (n = 24)	Shellfish (n = 1)	Mollusks (n = 8)	Crustacean (n = 1)
Tetracycline	3.2	4.2	0	0	0
Trimethoprim/sulfamethoxazole	0	12.5	0	0	0
Cefotaxime	9.7	12.5	0	12.5	0

### Detection of ARGs

ARGs were detected in a total of nine tetracycline-, trimethoprim/sulfamethoxazole-, and/or trimethoprim/sulfamethoxazole-resistant *Vibrio* and *Aeromonas* spp. isolates derived from seven (21.9%) fish samples (two wild-caught and five farm-raised) from six prefectures (**Table 4)**. The *tetD* gene was detected in the tetracycline-resistant *V. alginolyticus* isolate derived from wild-caught fish. In the tetracycline-resistant *V. anguillarum* isolate, derived from farm-raised fish, *tetB* and/or *tetM* genes were detected. In the sulfamethoxazole- and/or trimethoprim/sulfamethoxazole-resistant isolates, *sul1* and/or *sul2* genes were detected. Moreover, the *tetE* gene was detected in the tetracycline-resistant *A. veronii* and *A. caviae* isolates derived from wild-caught fish and farm-raised fish, respectively. In *A. caviae* isolates, derived from farm-raised fish, the *sul1* and *dfrA* genes were detected. The genes detected in our study have been frequently detected in various bacterial species derived from seafood^15^^,^^26^^,^^30^^)^, and are often transferable across genera^35^^)^. The *tetB* and *tetM* genes were specifically detected in isolates derived from farm-raised fish. A previous study demonstrated that the abundance of *tetB* and *tetM* genes in fish and aquatic environments increased after the application of oxytetracycline in aquaculture^36^^)^. These findings suggest that the antimicrobials use serves as a selective pressure for ARGs-carrying bacteria. Furthermore, farm-raised fish have been shown to contribute to ARGs enrichment in the sediments of aquaculture environments^37^^)^; thereby, posing a potential risk of their dissemination to the surrounding environments. Overall, antimicrobial usage in aquaculture practices may serve as a source of ARGs, and aquaculture-related ARGs may disseminate through various routes, including food chains and environments^5^^)^.

**Table 4.** Characteristics of antimicrobial resistance genes carrying *Vibrio* and *Aeromonas* isolates from raw seafood

Strain No	Sample No	Prefectures of origin	Sample type		Bacterial species	Antimicrobial resistant profiles	Antimicrobial resistance genes
52V-8	50	Hokkaido	Wild-caught	Trout fish	*V. alginolyticus*	Tetracycline	*tetD*
1V-10	1	Ehime	Farm-raised	Red sea bream	*V. anguillarum*	Sulfamethoxazole-, Trimethoprim/ sulfamethoxazole-Ampicillin	*sul2*
1V-1D	1	Ehime	Farm-raised	Red sea bream	*V. anguillarum*	Tetracycline-Ampicillin	*tetB*
45V-1	45	Ehime	Farm-raised	Red sea bream	*V. anguillarum*	Tetracycline-Sulfamethoxazole-, Trimethoprim/ sulfamethoxazole-Ampicillin	*tetB, tetM,sul1, sul2*
53V-7	53	Aomori	Wild-caught	Mackerel	*A. veronii*	Tetracycline	*tetE*
8V-5	8	Kochi	Farm-raised	Young yellowtail	*A. caviae*	Trimethoprim/sulfamethoxazole	*sul1, dfrA*
9V-3	9	Oita	Farm-raised	Greater amberjack	*A. caviae*	Trimethoprim/sulfamethoxazole	*sul1, dfrA*
C9V-5	9	Oita	Farm-raised	Greater amberjack	*A. caviae*	Trimethoprim/ sulfamethoxazole-Cefotaxime	*sul1, dfrA*
18V-9	18	Kagoshima	Farm-raised	Greater amberjack	*A. caviae*	Tetracycline	*tetE*

## Conclusion

As a pilot study for monitoring ARB in seafoods, our data showed that *Vibrio* spp. and *Aeromonas* spp. were isolated from more than 70% of raw seafood flesh samples in Japan, and that ARB-harboring ARGs (at least *tet*, *sul*, and *dfr* genes) were isolated in 21.9% of the fish samples. Notably, bacteria derived from farm-raised fish may be influenced by the use of antimicrobials in aquaculture. Given that seafoods are commonly consumed raw, ARB contamination in seafood could represent an important route of transmission to humans^9^^)^. As seafood consumption continues to increase, sustainably managing marine resources and controlling ARB in aquaculture farms to secure the stable and safe supply of seafood is essential^5^^)^. Therefore, ARB monitoring, particularly in water- and seafood-borne bacteria, such as *Vibrio* and *Aeromonas* spp., is required to ensure sustainable and safe provision of seafood.

## Supplementary materials

**Table S1.** Sample types of raw seafood

Fish (32; wild caught (14), farm raised (18))						Shellfish (26)	Mollusks (25)	Crustacean (2)
Perciformes		Clupeiformes	Pleuronectiformes		Gadiformes			
Wild caught	Farm raised	Wild caught	Wild caught	Farm raised	Wild caught	Wild caught	Wild caught	Wild caught
Yellowtail (1)	Yellowtail (3)	Trout fish (1)	Flounder (1)	Flounder (1)	Cod fish (1)	Scallops (14)	Octopus (14)	Shrimp (2)
Tuna (1)	Tuna (1)	Sardines (1)	Flatfish (1)			Shellfish (6)	Squid (11)	
Atka mackerel (2)	Red sea bream (7)					Surf clam (3)		
Mackerel (1)	Greater amberjack (3)					Oyster (2)		
Jack mackerel (1)	Young yellowtail (3)					Turban shell (1)		
Podothecus sachi (1)								

**Table S2.** Prefecture of origin of the seafood samples

Prefectures	Total (n = 85)	Wild-caught fish (n = 14)	Farm-raised fish (n = 18)	Shellfish (n = 26)	Mollusks (n = 25)	Crustacean (n = 2)
Hokkaido	61	10	0	25	24	2
Ehime	9	1	8	0	0	0
Oita	3	0	3	0	0	0
Nagasaki	3	0	3	0	0	0
Miyagi	2	2	0	0	0	0
Kumamoto	2	0	2	0	0	0
Aomori	2	1	0	0	1	0
Kochi	1	0	1	0	0	0
Kagoshima	1	0	1	0	0	0
Chiba	1	0	0	1	0	0

**Table S3.** Primer list for the identification of *Vibrio* and *Aeromonas* spp.

Bacteiral species or target genes	Prime name	Sequence	Annealing tempature (°C)	Amplicon size (bp)	Reference
*V. parahaemolyticus*	VP 1155272 F	AGCTTATTGGCGGTTTCTGTCGG	60	297	(Kim et al., 2015)
	VP 1155272 R	CKCAAGACCAAGAAAAGCCGTC			
*V. cholerae*	VC C634002 F	CAAGCTCCGCATGTCCAGAAGC	60	154	
	VC C634002 R	GGGGCGTGACGCGAATGATT			
*V. vulnificus*	W 2055918 F79	CAGCCGGACGTCGTCCATTTTG	60	484	
	W 2055918 R	ATGAGTAAGCGTCCGACGCGT			
*V. alginolyticus*	VA 1198230 F	ACGGCATTGGAAATTGCGACTG	60	199	
	VA 1198230 R	TACCCGTCTCACGAGCCCAAG			
*V. mimicus*	VMC727581F	ATAAAGCGGGGTGCGTGCA	60	249	
	VM C727581R	GATTTGGRAAAATCCKTCGTGC			
Genus of *Vibrio*	VG C2694352 F46	GTCARATTGAAAARCARTTYGGTAAAGG	60	689	
	VG C2694352 R734	ACYTTRATRCGNGTTTCRTTRCC			
Genus of *Vibrio*	Vuni-rpoD-F.4	CTTTYGCTTCACCGATAGACAT	60	1075	(Kim et al., 2019)
	Vuni-rpoD-R	CAAGGCTATCTGACCTACGC			
*V. anguillarum*	Van-rpoD-F.7	CAGACARCAAGAAGAAGACATTCG	60	125	
*V. scophthalmi*	Vsc-rpoD-F.2	CCAAGTTCAAAACGCCGTTGCA	60	740	
*V. lentus*	Vle-rpoD-F.3	GAACGGTAATCGTCGCTCAGTCC	60	113	
*V. alginolyticus*	Val-rpoD-F.2	AATGAAATGATGCTAGACGTATTCCG	60	371	
*V. gigantis*	Vgi-tox-R	GGCATGATGAAAGCGATAAGCAGT	60	292	
	Vuni-tox-F	CCWAARCGCGGTTAYCAAYTKAT			
*V. atlanticus*	Vat-recF-F.2	ATTTGAGAGTTCACTCGCGGGC	60	372	
	Vat-recF-F.3	GCTTAGTTCTCGATAGTGTGTTGC			
*V. harveyi*	VH-4F	GTGATGAAGAAGCTTATCGCGATT	60	601	
	VH-7R	CGCCTTCTTCAGTTAACGCAGGA			
*V. splendidus*	Vspl-tox-R.1	GTTGTTGCTGGTTCCACTTCAAC	60	218	
	Vuni-tox-F	CCWAARCGCGGTTAYCAAYTKAT			
*A. media*	A-med F(176)	GGCCAAGCGTCTGCGT	63	70	(Persson et al., 2015)
	A-med R(275)	CGCCCTCGTAGCAGAAGTGA			
*A. caviae*	A-cav F(4)	TGCTGCTGACCATCCGC	63	99	
	A-cav R(74)	GGTGCCTGCGGCTCG			
*A. hydrophila*	A-hyd F(533)	AGTCTGCCGCCAGTGGC	63	144	
	A-hyd R(677)	CRCCCATCGCCTGTTCG			
*A. veronii*	A-Ver F(b1)	CGTGCCGGCTTTGAAGTC	63	224	
	A-Ver R(b1-225)	GATCACGTACTTGCCTTCTTCAATA			
Genus of *Aeromonas*	A-16S F(270)	CGACGATCCCTAGCTGGTCT	63	461	
	A-16S R(731)	GCCTTCGCCACCGGTAT			
*lip*	Hydrolipase-F	AACCTGGTTCCGCTCAAGCCGTTG	55	760	(Sen, 2005)
	Hydrolipase-R	TTGCTCGCCTCGGCCCAGCAGCT			
*16S rRNA*	Schubertii-16S (UF-2)-F	TACTGGAAACGGTAGCT	55	322	
	Schubertii-16S (UF-2)-R	CTGGCAGGTATTAACCACCA			
*16S rRNA*	Popoffii-16S (UF-1)-F	AGTTGGAAACGACTGCT	55	323	
	Popoffii-16S (UF-1)-R	GTTGCTGGRTATTAGCCAA			
*ahyB*	Elastase-F2	ACACGGTCAAGGAGATCAAC	55	540	
	Elastase-R2	CGCTGGTGTTGGCCAGCAGG			
*gyrB*	gyrB-veronii-F	CCATTTTCAGCGATACCCTGTT	55	101	
	gyrB-veronii-R	CTCGTCTTGCAGACGGATGGAG			
*16S rRNA*	Jandaei-16S-F	TACTGGAAACGGTAGCT	55	322	
	Jandaei-16S (UR) R	CCAGCAGATATTAGCTACTG			
*16S rRNA*	Caviae-16S-F	AGTTGGAAACGACTGCT	55	322	
	Caviae-16S-R	CCAGCAGATATTAGCTACTG			
*pla/lip*	Lipase-F	ATCTTCTCCGACTGGTTCGG	55	383-389	
	Lipase-R	CCGTGCCAGGACTGGGTCTT			
*16S rRNA*	Veronii-16S-F	GAGGAAAGGTTGGTAGCTAATAA	55	688	
	Veronii-16S-R	CGTGCTGGCAACAAAGGACAG			
*gyrB*	gyrB-bestiarum-F	CACTTCTCCACAGAGCAGGAC	55	182	
	gyrB-bestiarum-R	ATCCTCTTTGTCCATATAGGT			
*gyrB*	gyrB-eucrenophila-F	AGCAGACTCGGCGACAGCTCT	55	177	
	gyrB-eucrenophila-R	CCTCGCGGCCATCGCGCTCG			

**Table S4.** Primer list for the detection of antimicrobial resistance genes

Antimcirobial resistance genes	Prime name	Sequence	Annealing tempature (°C)	Amplicon size (bp)	Reference
	tet-F	GCGCTNTATGCGTTGATGCA			(Kim et al., 2004)
*tetA*	tetA-R	ACAGCCCGTCAGGAAATT	55	387	
*tetB*	tetB-R	TGAAAGCAAACGGCCTAA	55	171	
*tetC*	tetC-R	CGTGCAAGATTCCGAATA	55	631	
*tetD*	tetD-R	CCAGAGGTTTAAGCAGTGT	55	484	
*tetE*	tetE-R	ATGTGTCCTGGATTCCT	55	246	
*tetG*	tetG-R	ATGCCAACACCCCCGGCG	55	803	
*tetM*	tetM-F	GTTAAATAGTGTTCTTGGAG	55	656	(H. J. Kim et al., 2015)
	tetM-R	CTAAGATATGGCTCTAACAA			
*tetS*	tetS-F	CATAGACAAGCCGTTGACC	56	667	
	tetS-R	ATGTTTTTGGAACGACAGAG			
*sul1*	Sul1-F	CGGCGTGGGCTACCTGAACG	59	433	(Phuong Hoa et al., 2008)
	Sul1-R	GCCGATCGCGTGAAGTTCCG			
*sul2*	Sul2-F	GCGCTCAAGGCAGATGGCATT	58	293	
	Sul2-R	GCGTTTGATACCGGCACCCGT			
*sul3*	Sul3-F	TCAAAGCAAAATGATATGAGC	54	787	
	Sul3-R	TTTCAAGGCATCTGATAAAGAC			
*sul4*	sul4_FW	CGCTTCATCGGGGTAAAAT	58	213	(Xu et al., 2020)
	sul4_RV	CGGACCTATTAAGATGGGAAA			
*dfrA1,A15,A16,A28*	dfr_group1-1f	GTGAAACTATCACTAATGG	46	471	(Šeputiene et al., 2010)
	dfr_group1-1r	ACCCTTTTGCCAGATTTG			
*dfrA8,A12,A13,A21,A22*	dfr_group1-2f	TTGGGAAGGACAACGCACTT	46	382	
	dfr_group1-2r	ACCATTTCGGCCAGATCAAC			
*dfrA12,A13,A21,A22*	dfr_group1-3f	GGTGAGCARAAGATYTTTCGC	46	309	
	dfr_group1-3r	TGGGAAGAAGGCGTCACCCTC			
*dfrA5,A14,A25,A27*	dfr_group2-1f	GCBAAAGGDGARCAGCT	44	394	
	dfr_group2-1r	TTTMCCAYATTTGATAGC			
*dfrA7,A17*	dfr_group2-2f	AAAATTTCATTGATTTCTGCA	44	471	
	dfr_group2-2r	TTAGCCTTTTTTCCAAATCT			
*dfrA3b*	dfr_group2-3f	TTTATTGTGGTAAGCAATAC	44	201	
	dfr_group2-3r	GTATACATCTGCATCAAAAC			
*dfrA3b*	dfr_group3-1f	ACCTGCCGATCTGCGTCAT	52	387	
	dfr_group3-1r	TCGCAGGCATAGCTGTTCTT			
*dfrA10*	dfr_group3-2f	ACCAGAGCATTCGGTAATCA	52	445	
	dfr_group3-2r	TTGGATCACCTACCCATAGA			
*dfrA26*	dfr_group3-3f	CACAGTCTATCGCCTTAATC	52	233	
	dfr_group3-3r	ATAGACCACAAAGCTAAACG			
*dfrA6*	dfr_group4-1f	GTTTCCGAGAATGGAGTAAT	52	429	
	dfr_group4-1r	GGTACGTGTAATCAATATTTG			
*dfrA24*	dfr_group4-2f	TCACCAAGAAGTCAGAGATT	52	311	
	dfr_group4-2r	TAAAACCAGATTCGACTTTC			
*dfrA23*	dfr_group4-3f	AGAATTCCCTTCTCTTTGAT	52	218	
	dfr_group4-3r	ATGCCAACAGTTGAGATTAT			
*dfrB1,B2,B3,B4,B5,B6*	dfr_group5-1f	GATCACGTRCGCAAGAARTC	56	95	
	dfr_group5-1r	GACTCGACVGCRTASCCTTC			
*dfrA9*	dfr_group5-2f	TGAACCAGAAGATTTAAAACAC	56	384	
	dfr_group5-2r	AATGGTCGGGACCTCAGAT			
*dfrA19*	dfr_group5-3f	AGTCGCTGTGGATTCTAAGT	56	455	
	dfr_group5-3r	CAATGTGAAAATTGTTCTGG			
*dfrA20*	dfr_group5-4f	ATGATTTGCTTTGGCACTTA	56	250	
	dfr_group5-4r	CCACCAATAATGAAGCATGT			

**Table S5.** Species of *Vibrio* and *Aeromonas* isolates from raw seafood

(n)	Total	Fish		Shellfish	Mollusks	Crustacean
		Wild-caught	Farm-raised			
Genus *Vibrio*	115	13	22	52	24	4
*V. alginolyticus*	81	10	6	45	18	2
*V. anguillarum*	14	0	9	5	0	0
*V. harvey*	6	0	3	1	0	2
other *Vibrio* spp.	14	3	4	1	6	0
Genus *Aeromonas*	65	31	24	1	8	1
*A. veronii*	24	8	11	0	5	0
*A. salmonicida*	15	11	2	1	1	0
*A. hydrophila*	9	4	4	0	0	1
*A. caviae*	6	2	4	0	0	0
*A. bestiarum*	4	4	0	0	0	0
*A. media*	3	0	1	0	2	0
*A. eucrenophila*	2	1	1	0	0	0
*A. enteropelogenes*	1	0	1	0	0	0
*A. popoffii*	1	1	0	0	0	0

## References

[r1] 1.Woolhouse M, Ward M, van Bunnik B, Farrar J. Antimicrobial resistance in humans, livestock and the wider environment. Philos Trans R Soc B Biol Sci. 2015; 370(1670): 20140083. . 10.1098/rstb.2014.0083 PMC4424433 25918441

[2] Sader HS, Castanheira M, Arends SJR, Goossens H, Flamm RK. Geographical and temporal variation in the frequency and antimicrobial susceptibility of bacteria isolated from patients hospitalized with bacterial pneumonia: results from 20 years of the SENTRY Antimicrobial Surveillance Program (1997–2016). J Antimicrob Chemother. 2019; 74(6): 1595–1606. . 10.1093/jac/dkz074 30843070

[3] NAOR. Nippon AMR One Health Report (NAOR). 2021. https://amr-onehealth.ncgm.go.jp/en/. Accessed April, 2023.

[4] Hossain A, Habibullah-Al-Mamun M, Nagano I, Masunaga S, Kitazawa D, Matsuda H. Antibiotics, antibiotic-resistant bacteria, and resistance genes in aquaculture: risks, current concern, and future thinking. Environ Sci Pollut Res Int. 2022; 29(8): 11054–11075. . 10.1007/s11356-021-17825-4 35028843

[5] Naylor RL, Hardy RW, Buschmann AH, et al. A 20-year retrospective review of global aquaculture. Nature. 2021; 591(7851): 551–563. . 10.1038/s41586-021-03308-6 33762770

[r6] 6.Kumar S, Lekshmi M, Parvathi A, Nayak BB, Varela MF. Antibiotic resistance in seafood-borne pathogens. Foodborne Pathog Antibiot Resist. 2017; **17**: 397–415. . 10.1002/9781119139188.ch17

[7] Harada T, Taguchi M, Kawahara R, Kanki M, Kawatsu K. Prevalence and antimicrobial susceptibility of bacterial pathogens in ready-to-eat foods retailed in Osaka prefecture, Japan. J Food Prot. 2018; 81(9): 1450–1458. . 10.4315/0362-028X.JFP-18-035 30080122

[8] Onjong HA, Ngayo MO, Mwaniki M, Wambui J, Njage PMK. Microbiological safety of fresh tilapia (*Oreochromis niloticus*) from Kenyan fresh water fish value chains. J Food Prot. 2018; 81(12): 1973–1981. . 10.4315/0362-028X.JFP-18-078 30457388

[9] Cabello FC, Godfrey HP, Tomova A, et al. Antimicrobial use in aquaculture re-examined: its relevance to antimicrobial resistance and to animal and human health. Environ Microbiol. 2013; 15(7): 1917–1942. . 10.1111/1462-2920.12134 23711078

[10] Zdanowicz M, Mudryk ZJ, Perliński P. Abundance and antibiotic resistance of *Aeromonas* isolated from the water of three carp ponds. Vet Res Commun. 2020; 44(1): 9–18. . 10.1007/s11259-020-09768-x 31965460 PMC7040064

[11] Mancini ME, Alessiani A, Donatiello A, et al. Systematic survey of *Vibrio* spp. and *Salmonella* spp. in Bivalve shellfish in Apulia region (Italy): prevalence and antimicrobial resistance. Microorganisms. 2023; 11(2): 450. . 10.3390/microorganisms11020450 36838415 PMC9966029

[12] Tan CW, Rukayadi Y, Hasan H, et al. Prevalence and antibiotic resistance patterns of *Vibrio parahaemolyticus* isolated from different types of seafood in Selangor, Malaysia. Saudi J Biol Sci. 2020; 27(6): 1602–1608. . 10.1016/j.sjbs.2020.01.002 32489301 PMC7253911

[13] Santajit S, Kong-ngoen T, Tunyong W, et al. Occurrence, antimicrobial resistance, virulence, and biofilm formation capacity of *Vibrio* spp. and *Aeromonas* spp. isolated from raw seafood marketed in Bangkok, Thailand. Vet World. 2022; 15(7): 1887–1895. . 10.14202/vetworld.2022.1887-1895 36185513 PMC9394122

[14] Elbashir S, Parveen S, Schwarz J, Rippen T, Jahncke M, DePaola A. Seafood pathogens and information on antimicrobial resistance: A review. Food Microbiol. 2018; 70: 85–93. . 10.1016/j.fm.2017.09.011 29173644

[15] Kumarage PM, De Silva LADS, Heo GJ. Aquatic environments: A potential source of antimicrobial-resistant Vibrio spp. J Appl Microbiol. 2022; 133(4): 2267–2279. . 10.1111/jam.15702 35797342

[16] Dierig A, Frei R, Egli A. The fast route to microbe identification: matrix assisted laser desorption/ionization-time of flight mass spectrometry (MALDI-TOF MS). Pediatr Infect Dis J. 2015; 34(1): 97–99. . 10.1097/INF.0000000000000601 25741802

[17] Kim HJ, Ryu JO, Lee SY, Kim ES, Kim HY. Multiplex PCR for detection of the Vibrio genus and five pathogenic Vibrio species with primer sets designed using comparative genomics. BMC Microbiol. 2015; 15(1): 239. . 10.1186/s12866-015-0577-3 26502878 PMC4624192

[18] Kim KI, Won K-M, Lee ES, Cho M, Jung SH, Kim MS. Detection of Vibrio and ten Vibrio species in cage-cultured fish by multiplex polymerase chain reaction using house-keeping genes. Aquaculture. 2019; 506: 417–423. . 10.1016/j.aquaculture.2019.03.073

[19] Persson S, Al-Shuweli S, Yapici S, Jensen JN, Olsen KEP. Identification of clinical *aeromonas* species by *rpoB* and *gyrB* sequencing and development of a multiplex PCR method for detection of *Aeromonas hydrophila, A. caviae, A. veronii*, and *A. media*. J Clin Microbiol. 2015; 53(2): 653–656. . 10.1128/JCM.01963-14 25411168 PMC4298543

[20] Sen K. Development of a rapid identification method for *Aeromonas* species by multiplex-PCR. Can J Microbiol. 2005; 51(11): 957–966. . 10.1139/w05-089 16333335

[21] CLSI. *Methods for Antimicrobial Dilution and Disk Susceptibility Testing of Infrequently Isolated or Fastidious Bacteria*. 3rd Edition. CLSI guidelines M45. Wayne, PA: Clinical and Laboratory Standards Institute; 2015.

[22] Kim SR, Nonaka L, Suzuki S. Occurrence of tetracycline resistance genes *tet* (M) and *tet* (S) in bacteria from marine aquaculture sites. FEMS Microbiol Lett. 2004; 237(1): 147–156. . 10.1111/j.1574-6968.2004.tb09690.x 15268950

[23] Šeputienė V, Povilonis J, Ružauskas M, Pavilonis A, Sužiedėlienė E. Prevalence of trimethoprim resistance genes in *Escherichia coli* isolates of human and animal origin in Lithuania. J Med Microbiol. 2010; 59(3): 315–322. . 10.1099/jmm.0.015008-0 20007760

[24] Phuong Hoa PT, Nonaka L, Hung Viet P, Suzuki S. Detection of the *sul1, sul2*, and *sul3* genes in sulfonamide-resistant bacteria from wastewater and shrimp ponds of north Vietnam. Sci Total Environ. 2008; 405(1-3): 377–384. . 10.1016/j.scitotenv.2008.06.023 18684492

[25] Xu F, Min F, Wang J, et al. Development and evaluation of a Luminex xTAG assay for sulfonamide resistance genes in *Escherichia coli* and *Salmonella* isolates. Mol Cell Probes. 2020; 49: 101476. . 10.1016/j.mcp.2019.101476 31678631

[26] De Silva LADS, Wickramanayake MVKS, Heo GJ. Virulence and antimicrobial resistance potential of *Aeromonas* spp. associated with shellfish. Lett Appl Microbiol. 2021; 73(2): 176–186. . 10.1111/lam.13489 33891720

[27] Ikeda-Ohtsubo W, Brugman S, Warden CH, Rebel JMJ, Folkerts G, Pieterse CMJ. How can we define “optimal microbiota?”: A comparative review of structure and functions of microbiota animals, fish, and plants in agriculture. Front Nutr. 2018; 5: 90. . 10.3389/fnut.2018.00090 30333981 PMC6176000

[28] Schar D, Klein EY, Laxminarayan R, Gilbert M, Van Boeckel TP. Global trends in antimicrobial use in aquaculture. Sci Rep. 2020; 10(1): 21878. . 10.1038/s41598-020-78849-3 33318576 PMC7736322

[29] Tate H, Ayers S, Nyirabahizi E, et al. Prevalence of antimicrobial resistance in select bacteria from retail seafood—United States, 2019. Front Microbiol. 2022; 13: 928509. . 10.3389/fmicb.2022.928509 35814688 PMC9262255

[30] Deng Y, Xu L, Chen H, et al. Prevalence, virulence genes, and antimicrobial resistance of *Vibrio* species isolated from diseased marine fish in South China. Sci Rep. 2020; 10(1): 14329. . 10.1038/s41598-020-71288-0 32868874 PMC7459350

[31] Lee HJ, Hoel S, Lunestad BT, Lerfall J, Jakobsen AN. *Aeromonas* spp. isolated from ready‐to‐eat seafood on the Norwegian market: prevalence, putative virulence factors and antimicrobial resistance. J Appl Microbiol. 2021; 130(4): 1380–1393. . 10.1111/jam.14865 33025711

[32] Hammad AM, Watanabe W, Fujii T, Shimamoto T. Occurrence and characteristics of methicillin-resistant and -susceptible *Staphylococcus aureus* and methicillin-resistant coagulase-negative staphylococci from Japanese retail ready-to-eat raw fish. Int J Food Microbiol. 2012; 156(3): 286–289. . 10.1016/j.ijfoodmicro.2012.03.022 22541390

[33] Hammad AM, Shimamoto T, Shimamoto T. Genetic characterization of antibiotic resistance and virulence factors in *Enterococcus* spp. from Japanese retail ready-to-eat raw fish. Food Microbiol. 2014; 38: 62–66. . 10.1016/j.fm.2013.08.010 24290627

[34] Amarasiri M, Sano D, Suzuki S. Understanding human health risks caused by antibiotic resistant bacteria (ARB) and antibiotic resistance genes (ARG) in water environments: Current knowledge and questions to be answered. Crit Rev Environ Sci Technol. 2020; 50(19): 2016–2059. . 10.1080/10643389.2019.1692611

[35] Domínguez M, Miranda CD, Fuentes O, et al. Occurrence of transferable integrons and suland dfr genes among sulfonamide- and/or trimethoprim-resistant bacteria isolated from chilean salmonid farms. Front Microbiol. 2019; 10: 748. . 10.3389/fmicb.2019.00748 31031727 PMC6474311

[36] Obayashi Y, Kadoya A, Kataoka N, et al. Tetracycline resistance gene profiles in red seabream (*Pagrus major*) intestine and rearing water after oxytetracycline administration. Front Microbiol. 2020; 11: 1764. . 10.3389/fmicb.2020.01764 32849389 PMC7417432

[37] Muziasari WI, Pitkänen LK, Sørum H, Stedtfeld RD, Tiedje JM, Virta M. The resistome of farmed fish feces contributes to the enrichment of antibiotic resistance genes in sediments below Baltic sea fish farms. Front Microbiol. 2017; 7: 2137. . 10.3389/fmicb.2016.02137 28111573 PMC5216021

